# First-episode psychosis intervention programme versus standard care for the clinical management of early phases of psychosis: cost analysis

**DOI:** 10.1192/bjo.2023.618

**Published:** 2023-12-22

**Authors:** Oliver Ibarrondo, María Recio-Barbero, Iker Ustarroz, Janire Cabezas-Garduño, Oihane Mentxaka, Teresa Acaiturri, Elisa Gómez, Rafael Segarra

**Affiliations:** RS-Statistics, Arrasate-Mondragón, Gipuzkoa, Spain; and Research Unit, Debagoiena Integrated Healthcare Organisation, Basque Health Service (Osakidetza), Arrasate-Mondragón, Guipúzcoa, Spain; Early Stages of Psychosis Group, Biocruces Bizkaia Health Research Institute, Barakaldo, Biscay, Spain; and Department of Neurosciences, University of the Basque Country (UPV/EHU), Leioa, Biscay, Spain; Economic-Financial Directorate, Cruces University Hospital, Barakaldo, Biscay, Spain; Early Stages of Psychosis Group, Biocruces Bizkaia Health Research Institute, Barakaldo, Biscay, Spain; and Department of Psychiatry, Cruces University Hospital, Barakaldo, Biscay, Spain; Early Stages of Psychosis Group, Biocruces Bizkaia Health Research Institute, Barakaldo, Biscay, Spain; Department of Neurosciences, University of the Basque Country (UPV/EHU), Leioa, Biscay, Spain; and Department of Psychiatry, Cruces University Hospital, Barakaldo, Biscay, Spain; Early Stages of Psychosis Group, Biocruces Bizkaia Health Research Institute, Barakaldo, Biscay, Spain; Department of Neurosciences, University of the Basque Country (UPV/EHU), Leioa, Biscay, Spain; Department of Psychiatry, Cruces University Hospital, Barakaldo, Biscay, Spain; and Centre for Biomedical Research in Mental Health, Carlos III Institute of Health (CIBERSAM ISCIII), Leioa, Biscay, Spain

**Keywords:** First-episode psychosis, psychotic disorders, schizophrenia, costs, economics

## Abstract

**Background:**

Early intervention programmes (EIPs) in psychosis have gained attention as specialised interventions to improve health-related and societal impacts for people with psychotic disorders. Previous studies have presented evidence in favour of EIPs over the first year of intervention, despite none considering the critical period before psychosis onset (5 years).

**Aims:**

To compare the associated costs of the First Episode Psychosis Intervention Program (CRUPEP) and treatment as usual (TAU) in a real-world cohort in a non-specialised psychiatric community setting.

**Method:**

Direct and indirect mental health-related costs were calculated over 1 year and up to 7 years. Healthcare and societal costs were calculated from economic data related to the consumption of all healthcare resources, including emergency department attendances, hospital admissions, psychotropic medication prescriptions and societal costs.

**Results:**

From a healthcare perspective, the intervention (CRUPEP) group initially showed a marginally higher cost per patient than the TAU group (€7621 TAU group *v.* €11 904 CRUPEP group) over the first year of follow-up. However, this difference was reversed between the groups on considering the entire follow-up, with the TAU group showing considerably higher associated costs per patient (€77 026 TAU *v.* €25 247 CRUPEP).

**Conclusions:**

The EIP (CRUPEP) showed clinical benefits and minimised the direct and indirect health-related costs of the management of psychosis. Although the CRUPEP intervention initially reported increased costs over 1 year, TAU surpassed the global costs over the entire follow-up.

Schizophrenia is a severe and enduring disease characterised by periods of symptom exacerbation or relapse. It has a low global incidence, with an estimated age-standardised prevalence of 0.28% worldwide.^1^ Despite gender differences in the onset of the disease, patients frequently present with a first psychotic episode during adolescence or early adulthood.^2^ Clinical trajectories before the first episode of psychosis (FEP) have been related to heterogeneous outcomes, predominantly functional disabilities and poor long-term prognosis.^3^ Thus, psychosocial dysfunction is a major factor that affects daily life and the ultimate achievement of functional recovery.

Psychosis is also associated with increased rates of medical comorbidities and mortality.^4–6^ In this regard, individuals with schizophrenia demonstrate nearly double the rates of smoking, obesity, hypertension and dyslipidaemia compared with the general population.^7^ In addition, the co-occurrence of chronic cardiovascular disorders, metabolic syndrome, diabetes and other psychiatric or neurological illnesses contributes to an increased overall burden of the disease.^8,9^ The type and number of comorbidities differ according to the gender.^10^

## Costs of schizophrenia treatment

The direct costs of schizophrenia account for up to 2.6% of total health expenditure in Western countries, which in turn accounts for 7–12% of gross national income. However, its multidimensional burden remains underestimated.^11^ The failure to estimate the actual burden of psychotic disorders accurately is largely attributed to indirect (i.e. lost productivity by patients and caregivers) rather than direct healthcare costs. Thus, the major economic burden of psychosis may be due to patient disability rather than the associated mortality and healthcare costs.^12–14^ In addition, social costs have been associated with lost productivity, informal care, the intervention of justice services and other social service costs.^12,14^ Despite its importance, the burden of informal care on family members remains underestimated, because informal care is varied and unpaid, provided by people with a direct social relationship with patients.

## Early intervention programmes

The health and socioeconomic impacts of schizophrenia pose a challenge for novel therapeutic paradigms. In this regard, the development of early intervention programmes (EIPs) for the detection and management of the early phases of psychosis has several advantages. The chief aims of EIPs include the early detection and treatment of psychotic symptoms, along with a reduction in the severity of illness and an improvement in long-term prognosis.^15^

Several studies have confirmed the cost-effectiveness of EIPs.^14,16,17^ Although some studies have suggested that EIPs incur a higher cost during the first 2 years of treatment, a gradual reduction is observed from the second year onwards, compared with standard care.^15^ However, there is limited evidence on the cost savings associated with treatment changes, because of the small number of EIP studies. Furthermore, indirect costs of early intervention management have a greater impact than the overall healthcare costs.^18^

In this study, we aimed to compare the healthcare costs of an EIP and standard care in first-episode psychosis. Furthermore, we intended to address a time horizon for the intervention's application covering the ‘critical period’ of the first 5 years of treatment and to analyse potential differences based on patients’ gender or major socioeconomic characteristics.

## Method

### Design

We performed a descriptive, retrospective and observational analysis to identify the primary differences between the First Episode Psychosis Intervention Program (CRUPEP) and standard community treatment for patients presenting with affective or non-affective FEP. The difference between the two programmes is based on the type of control performed. CRUPEP maintains a continuous follow-up on medication administration and constant health support, whereas in standard treatment patient control is the responsibility of patients themselves and/or caregivers. We identified and analysed costs (societal costs, both health and non-health) during the first year of treatment and throughout active follow-up between 2014 and 2020.

### Participants

All participants were patients with the Ezkerraldea Enkarterri Cruces Health Organisation (OSI-EEC), which covers a catchment area of 150 000 inhabitants from lower-middle socioeconomic urban districts located next to the greater Bilbao area in Basque Country, Spain. Potential participants were identified by ICD-10 coding (F20.X–F28.X; F31.2; F31.5; and F32.3), diagnosed between January 2014 and December 2019. The intervention group comprised individuals followed up by CRUPEP in the OSI-EEC's hospital (Cruces University Hospital, Barakaldo, Spain). The standard care (treatment as usual, TAU) group comprised patients attended by the primary community standard out-patient centres of the same OSI-EEC between 2014 and 2019. The inclusion criterion for both groups was being over 18 years of age. This is because CRUPEP only enrolled patients over this age.

### Intervention programme

CRUPEP offers intensive interdisciplinary follow-up care, combining medical treatment for psychosis and psychosocial counselling, to decrease the duration of untreated psychosis and to reduce its impact. The inclusion criterion was age >18 years. The exclusion criteria were: (a) prior antipsychotic treatment before psychosis onset, (b) the presence of organic brain disease and (c) IQ <70.

### Treatment as usual (TAU)

Standard community treatment consisted of standard psychiatric treatment offered by out-patient mental health services in the OSI-EEC catchment area. We considered patients with FEP who were not included in CRUPEP.

### Variables

Data for the study were obtained from Osakidetza's Oracle Analysis Service (OAS). The OAS is a database comprising anonymised administrative and clinical records from the Basque Health Service. The Economic Information System of Costs per Patient, implemented in the OSI-EEC facilitated the identification of accurate healthcare costs associated with each patient, thus enabling an assessment of the variability of costs derived from clinical practice as well as differences in resource use.

We extracted the primary sociodemographic and clinical variables from patients’ electronic medical records. The Economic Information System of Costs per Patient provides economic data related to the consumption of healthcare resources. The Osakidetza databases do not provide specific information on patients’ socioeconomic level. Therefore we used the pharmaceutical contribution code as a criterion to determine their socioeconomic level. The included variables are described in Supplementary Table 1, available at https://dx.doi.org/10.1192/bjo.2023.618.

The authors assert that all procedures contributing to this work comply with the ethical standards of the relevant national and institutional committees on human experimentation and with the Helsinki Declaration of 1975, as revised in 2008. All procedures involving human patients were approved by the Clinical Research Ethics Committee of Cruces University Hospital (CEIC code E20/21). All data were anonymised and therefore individual patient consent was not required.

#### Cost analysis

A cost analysis was conducted from two perspectives. We estimated the average costs of different factors for the intervention and TAU groups. In addition, we considered the following two time intervals: costs derived from the first year of treatment and costs over the entire follow-up. A description of each considered cost is given in Supplementary Table 2. Monetisation of social costs was not included owing to the high variability in labour unit costs; we included only the number of contacts that gave rise to a cost.

#### Statistical analyses

A descriptive analysis facilitated describing and determining the existence of statistically significant sociodemographic and clinical differences in variables, such as age, gender or socioeconomic level, through a univariate analysis. Fisher's exact test was performed for categorical variables with two categories and an expected value ≤5, whereas the chi-squared test was performed for continuous variables and an expected value >5. In addition, we estimated the comparison of means of continuous variables by applying analysis of variance when comparing more than three groups. Comparisons between two groups were performed using Student's *t*-test for normally distributed variables. Continuous variables without a normal distribution were analysed using the Kruskal–Wallis test to compare three or more groups and the Wilcoxon rank-sum test/Mann–Whitney test to compare two groups. Finally, the distribution of normality was assessed using non-parametric Kolmogorov–Smirnov tests.

Since participants’ initial characteristics were likely to be different for the two groups, propensity score analysis using a genetic matching algorithm was applied to ensure that both groups were comparable in terms of initial characteristics (age, gender, size of town of residence and income range).^19,20^ As Austin pointed out,^19^ using non-randomised studies to estimate the effects of treatments on outcomes we must take into account that in an observational design treatment selection is often influenced by individual characteristics. The balance process analysis is available in the Supplementary material.

The total cost analysis was performed using generalised linear models (GLMs). GLMs are a generalisation of least-squares linear regression that facilitate an analysis when the response variable does not follow a normal distribution.^21,22^ The analysis constructed GLMs with the total cost as dependent variable and participant characteristics as independent variables, with a significance level of 5%. Because participant follow-up could be variable, we considered the recorded follow-up time as a covariate of the model.

Statistical analysis was performed in several steps using the statistical programming software R (version 4.0.5 for Windows), with a confidence level of 95%.

## Results

Of the 252 patients who fulfilled the selection criteria, 30 were excluded because of the balance process. Table 1 summarises the sociodemographic data and contacts with the health system for the remaining 222 participants. The CRUPEP and TAU groups significantly differed in the sociodemographic variables, except for the gender distribution. Participants in the CRUPEP group were usually younger and residing in urban settings.

**Table 1:** Descriptive analysis of participants and contacts with the health system

Variable	TAU participants	CRUPEP participants	Total participants	*P*
Participants, *n*	91	131	222	
Gender, *n* (%)				
Female	31 (34.07%)	53 (40.46%)	84 (37.84%)	
Male	60 (65.93%)	78 (59.54%)	138 (62.16%)	0.377
Age group, years: , *n* (%)				
≤20	19 (20.88%)	45 (34.35%)	64 (28.83%)	
20–30	30 (32.97%)	45 (34.35%)	75 (33.78%)	
30–40	32 (35.16%)	25 (19.08%)	57 (25.68%)	
40–50	3 (3.30%)	0 (0.00%)	3 (1.35%)	
>50	7 (7.69%)	16 (12.21%)	23 (10.36%)	0.005
Place of residence (town size, thousands of inhabitants), *n* (%)				
<20	20 (21.98%)	24 (18.32%)	44 (19.82%)	
20–60	7 (7.69%)	5 (3.82%)	12 (5.41%)	
60–250	19 (20.88%)	53 (40.46%)	72 (32.43%)	
≥250	45 (49.45%)	49 (37.40%)	94 (42.34%)	0.016
Income range, thousand euros: *n* (%)				
<18	85 (93.41%)	126 (96.18%)	211 (95.05%)	
18–100	6 (6.59%)	5 (3.82%)	11 (4.95%)	0.364
Age, years: mean (s.d.)	33 (8.58)	33 (9.21)	33 (8.90)	0.6887
Follow-up, years: mean (s.d.)	7 (0.32)	5 (1.50)	6 (1.59)	<0.0001
Health system contacts^a^ over 1-year follow-up period, mean (s.d.)				
Emergencies	2 (2.18)	3 (2.96)	2 (2.70)	<0.0001
Emergencies with admission	1 (1.07)	1 (0.83)	1 (0.92)	0.0103
Emergencies without admission	2 (1.47)	2 (2.51)	2 (2.21)	0.1963
Acute general admissions	1 (1.07)	1 (0.87)	1 (1.00)	<0.0001
Day hospital	0 (0.99)	3 (11.27)	2 (8.15)	<0.0001
External consultations	0 (0.45)	10 (5.31)	5 (6.31)	<0.0001
Mental health external consultations	24 (37.90)	6 (8.21)	15 (28.87)	<0.0001
Mental health admissions	0 (0.45)	0 (0.18)	0 (0.34)	0.0006
Health system contacts^a^ over entire follow-up period, mean (s.d.)				
Emergencies	10 (11.81)	5 (4.80)	8 (9.35)	<0.0001
Emergencies with admission	3 (3.78)	2 (1.61)	2 (3.01)	<0.0001
Emergencies without admission	6 (9.08)	3 (3.57)	5 (7.15)	<0.0001
Acute general admissions	3 (3.98)	2 (1.95)	3 (3.21)	<0.0001
Day hospital	1 (7.00)	4 (11.33)	2 (9.48)	<0.0001
External consultations	0 (0.47)	21 (15.61)	11 (15.37)	<0.0001
Mental health external consultations	214 (216.40)	45 (80.17)	129 (183.55)	<0.0001
Mental health admissions	1 (1.63)	0 (0.31)	0 (1.22)	<0.0001

TAU, treatment as usual; CRUPEP, First Episode Psychosis Intervention Program.

a. Health system contacts are defined in Supplementary Table 1.

Overall, the number of contacts with the healthcare system depended on the study period. The CRUPEP group had a higher number of contacts with any part of the healthcare system during the first year of follow-up, whereas the TAU group had a higher cumulative number of such contacts over the entire follow-up (Table 1). A similar pattern was observed in attendances at an emergency department: the CRUPEP group had a higher average number of emergency department visits during the first year, but the TAU group experienced a significant increase in the number of emergency department attendances over the study.

Table 2 shows the estimated costs generated by the participants in both healthcare interventions (TAU and CRUPEP). Overall, the healthcare costs varied according to the length of the follow-up. Over the entire duration of the study, the average total cost per patient was €51 136; however, we observed differences depending on the period and type of intervention. During the first year, the average costs per patient for the TAU group and CRUPEP group were €7621 and €11 904 respectively. However, the TAU group participants had higher mean total costs over the entire follow-up (€77 026 for the TAU group versus €25 247 for the CRUPEP group).

**Table 2:** Health costs compared between interventions and separated by gender

	Costs (€) for CRUPEP participants				Costs (€) for TAU participants			
Variable	Total, mean (s.d.)	Female, mean (s.d.)	Male, mean (s.d.)	*P*	Total, mean (s.d.)	Female, mean (s.d.)	Male, mean (s.d.)	*P*
Health system contact^a^ over 1-year follow-up								
Emergency	655 (534.89)	756 (603.64)	602 (492.87)	0.0013	253 (406.71)	168 (363.98)	297 (423.27)	0.0006
Acute general admissions	7529 (7645.28)	9599 (6690.74)	6460 (7936.35)	<0.0001	2245 (4864.05)	1696 (4807.13)	2529 (4909.14)	0.0015
Day hospital	391 (1498.89)	44 (239.17)	570 (1817.16)	<0.0001	12 (102.25)	0 (0.00)	18 (125.85)	<0.0001
External consultations	936 (656.33)	1120 (713.81)	841 (609.14)	0.0050	1 (10.36)	0 (0.00)	2 (12.73)	0.0525
Mental health external consultations	628 (1024.82)	292 (528.40)	801 (1170.61)	0.0001	2249 (4096.26)	1443 (3442.52)	2666 (4364.83)	0.0043
Mental health admissions	163 (1352.96)	0 (0.00)	246 (1664.70)	0.0001	2275 (11 066.36)	1279 (7123.41)	2790 (12 657.71)	0.1825
Health system contact^a^ over entire follow-up								
Emergency	1057 (927.42)	1160 (1034.63)	1003 (871.33)	0.0301	1829 (1970.42)	1424 (1459.08)	2038 (2170.13)	0.0048
Acute general admissions	12 394 (14 142.81)	14 707 (13 284.43)	11 198 (14 529.78)	0.0006	15 242 (14 406.79)	14 286 (13 185.75)	15 735 (15 082.35)	0.7636
Day hospital	404 (1501.87)	47 (243.08)	588 (1819.13)	0.0001	97 (612.66)	0 (0.00)	148 (751.66)	<0.0001
External consultations	2388 (1889.44)	2777 (1699.92)	2187 (1963.55)	0.0043	6 (28.90)	9 (34.42)	5 (25.84)	0.7089
Mental health external consultations	3585 (7441.44)	1292 (2530.39)	4770 (8776.39)	0.0020	24 142 (30 313.64)	16 138 (14 952.26)	28 277 (35 171.10)	0.0014
Mental health admissions	196 (1458.71)	0 (0.00)	297 (1793.14)	0.0001	26 713 (109 787.71)	20 857 (103 506.08)	29 738 (113 629.14)	0.0340

TAU, treatment as usual; CRUPEP, First Episode Psychosis Intervention Program.

a. Health system contacts are defined in Supplementary Table 1.

Women in the CRUPEP group incurred higher costs than those in the TAU group during the first year. However, the opposite was seen for the men. The groups displayed a significant difference, except in the case of women admitted and followed-up at the out-patient mental health services (TAU group), with a similar distribution between the programmes. These differences between the groups were also significant for the entire follow-up. The CRUPEP group incurred higher healthcare costs for women and men during the first year of follow-up. However, these costs gradually balanced out between the groups, with the TAU group incurring higher costs in consultations and hospital admissions.

Similarly, the treatment prescriptions revealed differences by gender according to the time frame (Table 3). In this regard, anxiolytics and antidepressants were predominantly administered as adjunctive treatments during the first year in both treatment groups and genders. Overall, participants in the CRUPEP group had higher mean antipsychotic costs than those in the TAU group. However, as regards the distribution (the number of times a drug from each drug family was dispensed) for all classes of medication, those in the TAU group had a higher mean distribution and mean cost over the entire follow-up. Considering the entire follow-up, antipsychotic treatment incurred the highest costs. Participants in the TAU group had higher drug dispensing in all pharmacological groups, regardless of their gender.

**Table 3:** Dispensing and mean costs of out-patient drugs (balanced data)

	TAU participants		CRUPEP participants	
Variable	Distribution,^a^ mean (s.d.)	Cost (€), mean (s.d.)	Distribution,^a^ mean (s.d.)	Cost (€), mean (s.d.)
**Females**				
Over 1-year follow-up				
Anxiolytics	7 (10.04)	22 (46.65)	10 (10.94)	34 (43.11)
Anticholinergics	2 (1.94)	12 (12.15)	2 (0.00)	6 (0.00)
Antidepressants	12 (14.05)	245 (349.04)	10 (7.04)	157 (147.15)
Anticonvulsants	3 (2.45)	51 (89.25)	4 (5.23)	54 (64.43)
Antipsychotics	11 (8.67)	1059 (1364.81)	6 (4.30)	735 (1195.45)
Hypnotics/sedatives	6 (8.93)	19 (29.59)	6 (4.10)	24 (20.11)
Over entire follow-up				
Anxiolytics	15 (19.57)	46 (84.40)	43 (35.52)	190 (255.70)
Anticholinergics	3 (1.53)	12 (7.27)	15 (12.73)	64 (57.43)
Antidepressants	26 (24.49)	504 (525.46)	38 (32.79)	595 (637.95)
Anticonvulsants	8 (10.37)	94 (166.33)	19 (16.33)	250 (273.80)
Antipsychotics	36 (26.38)	4022 (4704.33)	57 (37.68)	6595 (7830.29)
Hypnotics/sedatives	12 (20.41)	41 (70.09)	21 (22.29)	90 (121.42)
**Males**				
Over 1-year follow-up				
Anxiolytics	9 (12.90)	33 (66.80)	13 (10.29)	39 (36.97)
Anticholinergics	5 (4.12)	26 (22.30)	3 (0.00)	10 (0.00)
Antidepressants	13 (19.00)	296 (452.83)	6 (4.06)	133 (179.21)
Anticonvulsants	9 (10.51)	255 (446.47)	10 (8.23)	356 (563.42)
Antipsychotics	15 (9.97)	1390 (1388.60)	10 (9.07)	1006 (1067.05)
Hypnotics/sedatives	5 (5.81)	18 (21.95)	7 (5.15)	31 (25.37)
Over entire follow-up				
Anxiolytics	18 (31.61)	88 (212.05)	36 (41.87)	147 (177.00)
Anticholinergics	4 (3.09)	18 (14.80)	5 (5.49)	24 (30.63)
Antidepressants	25 (34.96)	519 (1006.58)	31 (30.05)	550 (751.87)
Anticonvulsants	12 (20.88)	210 (463.03)	20 (33.25)	297 (724.98)
Antipsychotics	34 (30.12)	5238 (6025.09)	66 (56.15)	9558 (10143.72)
Hypnotics/sedatives	10 (10.29)	35 (43.29)	19 (21.13)	79 (91.57)

TAU, treatment as usual; CRUPEP, First Episode Psychosis Intervention Program.

a. Distribution refers to the number of times a drug from each drug family was dispensed at the pharmacy.

**Fig. 1:**
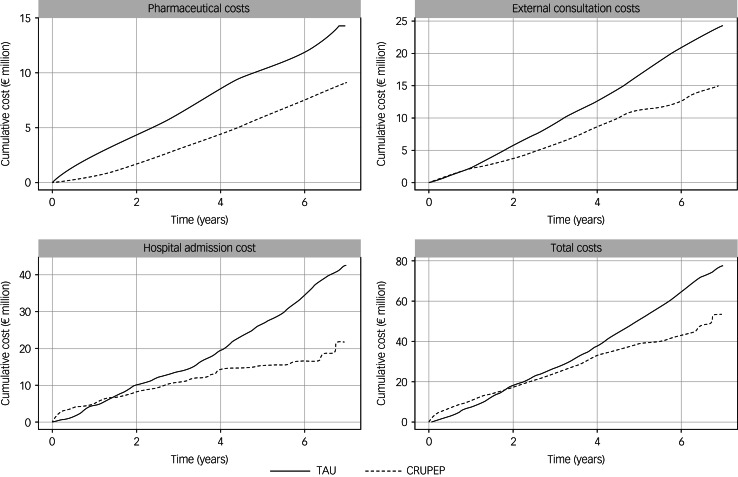
Cumulative costs over the entire follow-up period. TAU, treatment as usual; CRUPEP, First Episode Psychosis Intervention Program.

Figure 1 shows total costs in relation to the duration of follow-up. The CRUPEP group had a lower total cost per follow-up year than the TAU group. Despite higher costs for hospital admissions and consultations in the TAU group, the cost of prescribed psychotropic treatments was higher in the CRUPEP group. During the first 18–24 months, the CRUPEP group had a higher total cost. According to the GLMs (Supplementary Table 5), the overall cost was higher in the CRUPEP group during the first year, but the difference was statistically insignificant. By contrast, a cost analysis revealed that the CRUPEP group incurred significantly lower costs. In addition, participants’ socioeconomic status differentiated the total cost of treatment during the follow-up. The higher the socioeconomic status, the lower the total cost to the public health system.

The social impact of these groups was defined by productivity losses due to unemployment and hospital admission and by contacts with the legal system (Supplementary Table 6). The number of participants in work significantly differed between the groups, with a higher proportion in the CRUPEP group. Despite the age group, the TAU group had a higher number of participants receiving social benefits. However, the productivity loss per day spent in the hospital was comparable between the groups. The TAU group had a significantly higher mean number of legal proceedings associated with health status.

## Discussion

Our study presented evidence supporting the clinical benefits and cost reductions of treatment in a specialised early intervention unit for psychosis management over the critical period (i.e. the first 5 years). We assessed the primary differences according to the overall healthcare costs between the groups and period.

The average cost per patient was €51 136 over the entire duration of the study, although there were differences depending on the period and the type of intervention. Although the CRUPEP group initially reported increased costs during the first follow-up year, the global costs stabilised and increased over the years of follow-up in the TAU group.

Specifically, despite a marginal increase in the overall costs per patient for the CRUPEP group during the first year of treatment (€7621 for the TAU group and €11 904 for the CRUPEP group), the mean total cost per patient was notably higher for the TAU group after the complete follow-up (€77 026 for the TAU group *v.* €25 247 for the CRUPEP group). These results modestly differed from those reported by an early intervention programme (PAFIP) implemented in our neighbouring region, which had an estimated total cost per patient of €48 354 during the first year.^18^ The unitary costs of mental health external consultations depend on the type of contact or procedures performed. Thus, telephone contact costs are lower than face-to-face medical consultation costs. However, we did not differentiate the contact type because consultation type was not recorded in the records. This limitation resulted in overestimating the total cost, compared with the literature results. Nonetheless, the TAU total costs were higher than the CRUPEP total costs.

Considering follow-up periods >1 year, our results were similar to those reported in other cohorts. Over a 5-year follow-up in an Italian population, the estimated cost per patient of an EIP (€39 671) was lower than that of standard care.^15^ Likewise, Behan et al^23^ concluded that early intervention was cost-effective, costing €1681 less per patient over 1 year and involving fewer relapses. Other studies have reported that, despite higher treatment costs, EIP demonstrated cost-effectiveness as a consequence of the benefits achieved in patients’ quality of life.^14,24–26^

Beyond the economic implications, the benefits of early intervention programmes are reflected in clinical indicators, including the number of relapses. In our study, compared with the TAU group, the CRUPEP group required fewer in-patient admissions to both acute and medium-term care facilities over the 5-year follow-up.

Among the social costs, the loss of productivity resulting from hospital admission is a relevant variable, even though many people with psychotic illnesses are not in the workforce. The proportion of people with schizophrenia in employment has been estimated to be as high as 30% in some countries; however, it declines to 15% in Spain.^27^ In addition, the costs attributed to lost productivity have been estimated to be 60% of the indirect costs.^8^ Our results demonstrated a distinct difference in favour of the participants in the CRUPEP group. The CRUPEP group comprised a higher proportion of employed patients not receiving social benefits. The average admission times resulted in similar productivity losses; however, the cost of lost productivity should be interpreted cautiously because of the small percentage of patients in the labour force.^27^

From a societal perspective, social deprivation has been widely considered to be a risk factor for mental disorders, particularly schizophrenia.^28^ In our study, the average healthcare costs were higher among participants with lower incomes. The income level of people with severe mental illness determines the availability of professionalised non-healthcare interventions, like vocational therapy, that improves their adherence to treatment and prevents psychotic exacerbations, thereby reducing the need for healthcare.^27^

The proportion of gross domestic product per capita expended on the social costs of schizophrenia ranges from 37% (Switzerland) to 214% (the UK), depending on the country.^13^ In Spain, half of the social costs for people with schizophrenia are related to informal care.^29^ However, the quantification of social costs is a weakness of our study because of numerous social effects of schizophrenia, and, particularly, because several social costs are born by the family. It is difficult to identify factors such as productivity losses, informal care or the number of court cases related to patients from retrospective studies owing to the protection of personal data. However, recording the number of admissions following a court decision is a substitute for the size of social burden.

### Clinical implications

To promote early detection following onset of psychosis, the implementation of early intervention units has gained acceptance worldwide. The key features of early intervention include the specialised and sensitive management of the early stages of psychosis, prompting treatment adherence, reducing the risk of relapse and reducing the impact on quality of life and psychosocial functioning.^17,30^ The analysis of EIPs has demonstrated their cost reduction across diverse global FEP cohorts; nonetheless, their implementation in Spain remains challenging. The shortage of public funding is one of the chief barriers to their implementation.^31^ Thus, disparity in the access to specialised programmes in Spain has increased across the country. Policymakers should consider the key benefits offered by specialised mental health units according to the accumulated body of scientific research.

### Strengths and limitations

The chief contributions of our study included performing a cost analysis of an FEP programme in the first year, over a 5-year follow-up and up to 7 years. Furthermore, in accordance with international practice guidelines, researchers are required to provide disaggregated results according to the gender. Our results revealed differences in the profile of users according to their gender, which may influence clinical practice. Second, previous studies reported on external validity issues caused by restrictions on sample age. To overcome these limitations, we also included older patients with FEP. The need to enrol middle-aged patients (i.e. patients with a first episode when aged >45 years) has been highlighted, particularly because it may influence the analysis of real-world data from clinical practice. Our FEP programme did not have an upper age limit. This is because the mid-40s are a critical period for the onset of the second peak of affective and non-affective psychotic disorders.^32^

The methodological approach we used to estimate direct and indirect costs had potential limitations. First, real-world data analysis may include bias because, for many reasons, the compared populations may not be equivalent. Second, the estimation of the societal and healthcare burden of psychosis remains questionable, thus necessitating a major review. Access to databases across a number of separate organisations, including the social services department, was one of the primary difficulties encountered in this estimation. Third, we encountered methodological differences between FEP programmes in accounting for direct and indirect costs. Potential explanations for such differences include the overall challenges in estimating and comparing direct and indirect costs, with indirect costs being of particular relevance as they account for a high percentage of the total disease burden. Indirect social costs may pose a challenge to easily computing direct healthcare costs (e.g. hospital admissions, medical prescriptions) from a clinic management perspective. Such a situation poses a potential limitation in comparing EIPs for psychosis. Nevertheless, we provided an approximation of the indirect costs according to related parameters that may exert an effect on psychosocial functioning, including employment status, social deprivation and the number of involuntary hospital admissions.

## Supporting information

Ibarrondo et al. supplementary material Ibarrondo et al. supplementary material

## Data Availability

The data that support the findings of this study are available from the corresponding author, M.R.-B., on reasonable request.
