# Supplementary material for: First-episode psychosis intervention programme versus standard care for the clinical management of early phases of psychosis: cost analysis
Source: BJPsych Open. 2023 Dec 22;10(1):e17. doi: 10.1192/bjo.2023.618 (PMC10755560; doi:10.1192/bjo.2023.618)
Supplement: Ibarrondo et al. supplementary material [file S205647242300618Xsup001.docx]

**Supplementary material.**

**Genetic matching results**

The results of the matchig procedure can be measured through different indicators, and they determine if there is balance in the pre‐ and/or post‐matching datasets. Two univariate tests are usual: the t‐test and the bootstrap Kolmogorov‐Smirnov (KS) test. These tests should not be treated as hypothesis tests as usual because the objective tries to maximize balance without limit. The bootstrap KS test results are highly recommended because the bootstrap KS is consistent even for non‐continuous distributions.

The null hypothesis for the KS test analyzed equal balance in the estimated probabilities between treated and control. If the covariates being considered are discrete, this KS test is asymptotically nonparametric as long as the logit model does not produce zero parameter estimates.

The results of the tests before and after matching are shown in the following table.

| **Variable** |  | **Before Matching** | **After Matching** |
| --- | --- | --- | --- |
| Sex | mean treatment | 0.6273 | 0.6593 |
|  | mean control | 0.6593 | 0.6593 |
|  | std mean diff | -6.6000 | 0.0000 |
|  | Mean raw eQQ difference | 0.0330 | 0.0000 |
|  | Median raw eQQ difference | 0.0000 | 0.0000 |
|  | Max raw eQQ difference | 1.0000 | 0.0000 |
|  | Mean raw eCDF difference | 0.0160 | 0.0000 |
|  | Median raw eCDF difference | 0.0160 | 0.0000 |
|  | Max raw eCDF difference | 0.0320 | 0.0000 |
|  | Ratio (Tr/Co) | 1.0359 | 1.0000 |
|  | T-test p-value | 0.6114 | 1.0000 |
|  | KS Bootstrap p-value | - | - |
| Age | mean treatment | 35.1757 | 37.1109 |
|  | mean control | 37.2419 | 37.2419 |
|  | std mean diff | -18.0298 | -1.3616 |
|  | Mean raw eQQ difference | 3.3734 | 1.0112 |
|  | Median raw eQQ difference | 3.6073 | 0.8027 |
|  | Max raw eQQ difference | 19.0000 | 17.7479 |
|  | Mean raw eCDF difference | 0.0874 | 0.0297 |
|  | Median raw eCDF difference | 0.1065 | 0.0201 |
|  | Max raw eCDF difference | 0.1591 | 0.0914 |
|  | Ratio (Tr/Co) | 1.5710 | 1.1077 |
|  | T-test p-value | 0.1181 | 0.8116 |
|  | T-test p-value | 0.1000 | 0.0190 |
| Place of residence (town size) | mean treatment | 2.2547 | 2.4286 |
|  | mean control | 2.4286 | 2.4286 |
|  | std mean diff | -21.7223 | 0.0000 |
|  | Mean raw eQQ difference | 0.2527 | 0.0000 |
|  | Median raw eQQ difference | 0.0000 | 0.0000 |
|  | Max raw eQQ difference | 1.0000 | 0.0000 |
|  | Mean raw eCDF difference | 0.0633 | 0.0000 |
|  | Median raw eCDF difference | 0.0366 | 0.0000 |
|  | Max raw eCDF difference | 0.1801 | 0.0000 |
|  | Ratio (Tr/Co) | 0.7562 | 1.0000 |
|  | T-test p-value | 0.1334 | 1.0000 |
|  | T-test p-value | 0.0070 | 1.0000 |
| Income Range | mean treatment | 1.1925 | 1.0659 |
|  | mean control | 1.0659 | 1.0659 |
|  | std mean diff | 30.8039 | 0.0000 |
|  | Mean raw eQQ difference | 0.1319 | 0.0000 |
|  | Median raw eQQ difference | 0.0000 | 0.0000 |
|  | Max raw eQQ difference | 1.0000 | 0.0000 |
|  | Mean raw eCDF difference | 0.0422 | 0.0000 |
|  | Median raw eCDF difference | 0.0062 | 0.0000 |
|  | Max raw eCDF difference | 0.1204 | 0.0000 |
|  | Ratio (Tr/Co) | 2.7130 | 1.0000 |
|  | T-test p-value | 0.0026 | 1.0000 |
|  | T-test p-value | 0.0050 | 1.0000 |

The balance adjustment of the variables between controls and treated patients can be checked graphically (i.e., Program vs TAU). The representation is more visual with continuous variables. The adjustment of the continuous variable considered in the balancing process is shown below (Age of the patients).


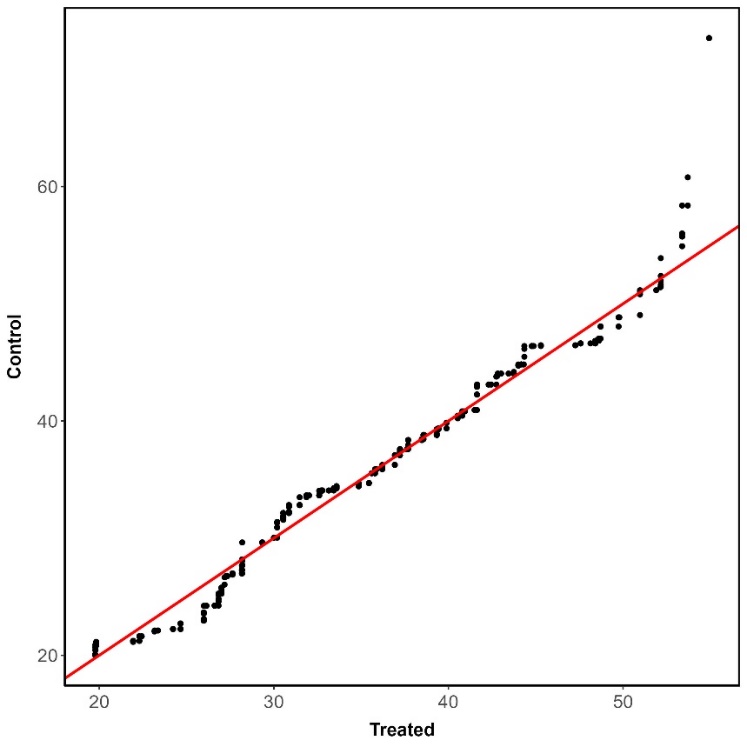


**Table S1.** Definition of the cost units considered.

| **Variables** | **Description** |
| --- | --- |
| **Welfare:** |  |
| Emergencies | Admissions to the emergency department in acute hospitals in the Basque Health Service network. Emergencies are not discriminated by service. |
| Emergencies with hospitalization | Admissions to the emergency department that lead to admission to an acute hospital of the Basque Health Service. Emergencies are not discriminated by service. |
| Emergencies without hospitalization | Admissions to the emergency department that do NOT lead to admission to an acute hospital of the Basque Health Service. Accident and emergency departments are not discriminated by service. |
| Acute general hospitalizations | Programmed hospitalisations in acute hospital of the Basque Health Service that do not come from an emergency episode. Emergencies are not discriminated by admission service. |
| Day hospital | Admissions to the medical day hospitalisation service in an acute care centre of the Basque Health Service. Medical day hospitalisation corresponds to interventions requiring hospital supervision with an interval between admission and discharge of less than 24 hours. |
| External consultations | Patient contacts for consultation with the psychiatry service of the acute hospital network. |
| Mental health external consultations | Patient contacts for consultations with the mental health service of the primary care service. Within the Basque Health Service, the mental health service is independent of the hospital psychiatry service. |
| Mental health hospitalizations | Medium-long stay admissions to the mental health service. Within the Basque health service, the medium-long stay centres are independent of the psychiatry service in acute hospitalisation. |
| **Pharmacological:** |  |
| Anxiolytics | Drugs belonging to the ATC code family N05B*. |
| Anticholinergics | Drugs belonging to the ATC code family N04A*. |
| Antidepressants | Drugs belonging to the ATC code family N06A*. |
| Anticonvulsants | Drugs belonging to the ATC code family N03A*. |
| Antipsychotic | Drugs belonging to the ATC code family N05A*. |
| Hypnotics / Sedatives | Drugs belonging to the ATC code family N05C*. |

**Table S2.** Descriptive analysis of patients and contacts with the health system.

| **Dimension** | **Cost Variable** | **Description** |
| --- | --- | --- |
| Health related costs | Emergencies | Patient admission in the acute emergency unit of a general hospital. |
|  | Emergencies with hospitalization | Patient admission in general acute emergency unit and discharge reason is a hospital admission. |
|  | Emergencies without hospitalization | Patient admission in general acute emergency unit and patient leave to their habitual residence. |
|  | Acute general hospitalizations | Patient admission to a general hospital, not a mental health hospital. |
|  | Day hospital | Patient admission to the day hospital service, a health care center where the patient receives the therapeutic techniques he needs without leaving his family environment. Once the procedures are finished, the patient returns home. |
|  | External consultations | Contacts with specialized health care on an outpatient basis for the diagnosis, treatment or follow-up of a patient. These consultations are different from primary care and do not include specialized mental health care. |
|  | Mental health external consultations | Contacts with healthcare specialized in mental health. |
|  | Mental health hospitalizations | Admissions to a specialized mental health hospital. |
| Social costs | Productivity loose | Productivity losses were calculated as the number of days that the patient was admitted to a hospital (mental health or not). Contacts with external consultations were not included. The cost monetization was not included due to the high variability in labour unit costs. The number of contacts were included. |
|  | Contac with legal service | Contacts with the health system derived from problems of a legal nature. The cost monetization was not included due to the high variability in legal unit costs. The number of contacts were included. |

**Table S3.** Descriptive analysis of patients and contacts with the health system (original raw data).

| **Variable** | **Values** | **TAU N (%)** | **Programme N (%)** | **Total N (%)** | **p.value** |
| --- | --- | --- | --- | --- | --- |
| N |  | 91 | 161 | 252 |  |
| Sex | Female | 31 (34.07%) | 60 (37.27%) | 91 (36.11%) |  |
|  | Male | 60 (65.93%) | 101 (62.73%) | 161 (63.89%) | 0.683 |
| Age group (years) | <=20 | 3 (3.30%) | 8 (4.97%) | 11 (4.37%) |  |
|  | (20-30] | 19 (20.88%) | 54 (33.54%) | 73 (28.97%) |  |
|  | (30-40] | 30 (32.97%) | 50 (31.06%) | 80 (31.75%) |  |
|  | (40-50] | 32 (35.16%) | 27 (16.77%) | 59 (23.41%) |  |
|  | >50 | 7 (7.69%) | 22 (13.66%) | 29 (11.51%) | 0.008 |
| Place of residence (town size, thousands of inhabitants) | <20 | 20 (21.98%) | 29 (18.01%) | 49 (19.44%) |  |
|  | 20-60 | 19 (20.88%) | 69 (42.86%) | 88 (34.92%) |  |
|  | 60-250 | 45 (49.45%) | 56 (34.78%) | 101 (40.08%) |  |
|  | >=250 | 7 (7.69%) | 7 (4.35%) | 14 (5.56%) | 0.005 |
| Income Range (thousands of euros) | <18 | 85 (93.41%) | 131 (81.37%) | 216 (85.71%) |  |
|  | 18-100 | 6 (6.59%) | 29 (18.01%) | 35 (13.89%) |  |
|  | >100 | 0 (0.00%) | 1 (0.62%) | 1 (0.40%) | 0.003 |
|  |  | **Mean (SD)** | **Mean (SD)** | **Mean (SD)** | **p.value** |
| Age (years) |  | 37 (9.14) | 35 (11.46) | 36 (10.71) | 0.0419 |
| Follow-up (years) |  | 7 (0.25) | 5 (1.43) | 5 (1.62) | 0.0000 |
|  |  | **Nº of events**  **Mean (SD)** | **Nº of events Mean (SD)** | **Nº of events Mean (SD)** | **p.value** |
| **1 year follow-up period** |  |  |  |  |  |
| Emergencies |  | 1 (2.02) | 3 (3.32) | 2 (3.02) | 0.0000 |
| Emergencies with hospitalization |  | 1 (0.96) | 1 (0.78) | 1 (0.82) | 0.2984 |
| Emergencies without hospitalization |  | 2 (1.57) | 2 (2.95) | 2 (2.72) | 0.5920 |
| Acute general hospitalizations |  | 0 (0.90) | 1 (0.88) | 1 (0.92) | 0.0000 |
| Day hospital |  | 0 (0.74) | 3 (16.40) | 2 (13.20) | 0.0000 |
| External consultations |  | 0 (0.52) | 10 (5.58) | 7 (6.61) | 0.0000 |
| Mental health external consultations |  | 21 (35.28) | 5 (7.82) | 11 (23.28) | 0.0001 |
| Mental health hospitalizations |  | 0 (0.38) | 0 (0.16) | 0 (0.26) | 0.1075 |
| **Complete follow-up period** |  |  |  |  |  |
| Emergencies |  | 8 (9.75) | 5 (4.92) | 6 (7.23) | 0.0039 |
| Emergencies with hospitalization |  | 3 (2.76) | 1 (1.40) | 2 (2.07) | 0.0001 |
| Emergencies without hospitalization |  | 5 (8.01) | 3 (4.11) | 4 (5.93) | 0.3092 |
| Acute general hospitalizations |  | 3 (2.88) | 2 (1.70) | 2 (2.25) | 0.0000 |
| Day hospital |  | 1 (7.91) | 4 (16.43) | 3 (14.00) | 0.0000 |
| External consultations |  | 0 (0.57) | 24 (18.73) | 16 (19.00) | 0.0000 |
| Mental health external consultations |  | 203 (225.40) | 27 (53.94) | 90 (165.25) | 0.0000 |
| Mental health hospitalizations |  | 1 (1.42) | 0 (0.21) | 0 (0.90) | 0.0000 |

**Table S4.** Health costs compared between interventions and separated by sex (original raw data).

|  | **Programme** | | | | **TAU** | | | |
| --- | --- | --- | --- | --- | --- | --- | --- | --- |
| **Variable** | **Total**  **Mean (SD)** | **Female**  **Mean (SD)** | **Male**  **Mean (SD)** | **p.value** | **Total**  **Mean (SD)** | **Female**  **Mean (SD)** | **Male**  **Mean (SD)** | **p.value** |
| **1 year follow-up** |  |  |  |  |  |  |  |  |
| Emergency | 631 (567.78) | 749 (731.96) | 560 (431.63) | 0.0200 | 253 (406.71) | 168 (363.98) | 297 (423.27) | 0.0595 |
| Acute general hospitalizations | 7,255 (8,077.15) | 8,747 (7,506.39) | 6,369 (8,307.30) | 0.0122 | 2,245 (4,864.05) | 1,696 (4,807.13) | 2,529 (4,909.14) | 0.0959 |
| Day hospital | 321 (1,433.56) | 101 (396.81) | 453 (1,774.43) | 0.0328 | 12 (102.25) | 0 (0.00) | 18 (125.85) | 0.3146 |
| External consultations | 825 (549.42) | 958 (590.35) | 746 (510.42) | 0.0380 | 1 (10.36) | 0 (0.00) | 2 (12.73) | 0.3146 |
| Mental health external consultations | 504 (820.89) | 386 (648.15) | 574 (903.90) | 0.6658 | 2,249 (4,096.26) | 1,443 (3,442.52) | 2,666 (4,364.83) | 0.1230 |
| Mental health hospitalizations | 232 (1,633.06) | 0 (0.00) | 370 (2,053.18) | 0.1213 | 2,275 (11,066.36) | 1,279 (7,123.41) | 2,790 (12,657.71) | 0.5042 |
| **Complete follow-up** |  |  |  |  |  |  |  |  |
| Emergency | 1,058 (982.10) | 1,200 (1,196.90) | 974 (823.82) | 0.1265 | 1,829 (1,970.42) | 1,424 (1,459.08) | 2,038 (2,170.13) | 0.0786 |
| Acute general hospitalizations | 11,697 (13,701.84) | 12,819 (12,407.38) | 11,031 (14,434.54) | 0.0830 | 15,242 (14,406.79) | 14,286 (13,185.75) | 15,735 (15,082.35) | 0.8505 |
| Day hospital | 337 (1,436.02) | 106 (399.74) | 473 (1,776.10) | 0.0351 | 97 (612.66) | 0 (0.00) | 148 (751.66) | 0.1471 |
| External consultations | 2,297 (1,791.90) | 2,808 (1,672.20) | 1,993 (1,799.30) | 0.0014 | 6 (28.90) | 9 (34.42) | 5 (25.84) | 0.7666 |
| Mental health external consultations | 2,897 (6,261.28) | 1,349 (2,506.82) | 3,817 (7,531.62) | 0.2863 | 24,142 (30,313.64) | 16,138 (14,952.26) | 28,277 (35,171.10) | 0.0815 |
| Mental health hospitalizations | 249 (1,678.72) | 0 (0.00) | 397 (2,109.39) | 0.1213 | 26,713 (109,787.71) | 20,857 (103,506.08) | 29,738 (113,629.14) | 0.2495 |

SD: Standard deviation

**Table S5.** Regression models for estimating total cost with balanced and raw data.

|  | **1 year follow-up** | | | **Complete follow-up** | | |
| --- | --- | --- | --- | --- | --- | --- |
| **Variable** | **Estimate** | **CI** | **Sig** | **Estimate** | **CI** | **Sig** |
| **Models constructed with balanced data** |  |  |  |  |  |  |
| (Intercept) | 9.102 | [8.71;9.49] | *** | 11.034 | [10.46;11.61] | *** |
| Group (Programme) | 0.229 | [0.08;0.38] | ** | -0.784 | [-0.99;-0.58] | *** |
| Sex (Male) | 0.053 | [-0.11;0.22] |  | 0.078 | [-0.07;0.22] |  |
| Income Range (18-100 m€) | -1.260 | [-1.56;-0.94] | *** | -0.763 | [-1.02;-0.49] | *** |
| Town size (20-60m inhabitants) | 0.215 | [-0.01;0.44] | . | -0.192 | [-0.39;0] | . |
| Town size (60-250m inhabitants) | 0.222 | [0.03;0.41] | * | -0.171 | [-0.34;-0.01] | * |
| Town size (>=250m inhabitants) | -0.058 | [-0.37;0.27] |  | -0.307 | [-0.58;-0.03] | * |
| Age | -0.002 | [-0.01;0.01] |  | -0.015 | [-0.02;-0.01] | *** |
| Follow-up (years) |  |  |  | 0.122 | [0.06;0.19] | *** |
| **Models constructed with raw data** |  |  |  |  |  |  |
| (Intercept) | 9,161 | [8.47 ; 9.86] | *** | 11,336 | [10.29 ; 12.39] | *** |
| Group (Programme) | 0,210 | [-0.13 ; 0.54] |  | -0,962 | [-1.35 ; -0.56] | *** |
| Sex (Male) | 0,031 | [-0.29 ; 0.35] |  | 0,068 | [-0.22 ; 0.36] |  |
| Income Range (18-100 m€) | -0,421 | [-0.84 ; 0.03] | . | -0,394 | [-0.77 ; 0.01] | * |
| Income Range (>100 m€) | -2,229 | [-4.09 ; 1.20] | . | -2,699 | [-4.44 ; 0.44] | * |
| Town size (20-60m inhabitants) | 0,030 | [-0.39 ; 0.43] |  | -0,314 | [-0.70 ; 0.06] | . |
| Town size (60-250m inhabitants) | 0,240 | [-0.18 ; 0.64] |  | -0,139 | [-0.52 ; 0.22] |  |
| Town size (>=250m inhabitants) | -0,205 | [-0.94 ; 0.65] |  | -0,416 | [-1.04 ; 0.29] |  |
| Age | -0,002 | [-0.02 ; 0.01] |  | -0,011 | [-0.02 ; 0.00] | . |
| Follow-up (years) |  |  |  | 0,064 | [-0.06 ; 0.18] |  |

**Table S6.** Social costs over the whole period (balanced data).

| **Variable** |  | **TAU N (%)** | **Programme N (%)** | **P value** |
| --- | --- | --- | --- | --- |
| **Work related activity** |  |  |  |  |
| Relationship with work | Active | 48 (52.75%) | 94 (71.76%) |  |
|  | Active - Social assistance recipient | 43 (47.25%) | 36 (27.48%) |  |
|  | Pensioner | 0 (0.00%) | 1 (0.76%) | 0.0020 |
|  |  | **Mean (SD)** | **Mean (SD)** | **P value** |
| Acute general hospitalizations time |  | 15.23 (9.92) | 15.75 (7.80) | 0.4996 |
| Mental health hospitalizations time |  | 63.53 (126.77) | 28.50 (-) | - |
| **Mental health related legal interventions** |  |  |  |  |
| Number of judgments |  | 2.62 (1.98) | 1.03 (0.17) | 0.0000 |

The SD of mental health hospitalization time could not be established in program patients because it only was two cases with equal weight.
